# Supplementary material for: Identification and functional analysis of non-coding regulatory small RNA FenSr3 in Bacillus amyloliquefaciens LPB-18
Source: PeerJ. 2023 May 15;11:e15236. doi: 10.7717/peerj.15236 (PMC10194069; doi:10.7717/peerj.15236)
Supplement: Supplemental Information 4 [file peerj-11-15236-s004.zip › KO/CK-vs-T1_map/map04640.html]

KEGG PATHWAY: Hematopoietic cell lineage - Reference pathway


|  |  |
| --- | --- |
| **Hematopoietic cell lineage - Reference pathway** |  |

[
Pathway menu
| Organism menu
| Pathway entry
| Show description
| User data mapping
]

Blood-cell development progresses from a hematopoietic stem cell (HSC), which can undergo either self-renewal or differentiation into a multilineage committed progenitor cell: a common lymphoid progenitor (CLP) or a common myeloid progenitor (CMP). A CLP gives rise to the lymphoid lineage of white blood cells or leukocytes-the natural killer (NK) cells and the T and B lymphocytes. A CMP gives rise to the myeloid lineage, which comprises the rest of the leukocytes, the erythrocytes (red blood cells), and the megakaryocytes that produce platelets important in blood clotting. Cells undergoing these differentiation process express a stage- and lineage-specific set of surface markers. Therefore cellular stages are identified by the specific expression patterns of these genes.

|  |  |  |
| --- | --- | --- |
| Reference pathway | 184% 150% 122% 100% 82% 67% 55% | 图片下载 |
